# Dr. Crane, on Mr. Cribb's Case of Impregnation

**Published:** 1805-11-01

**Authors:** William Crane

**Affiliations:** Boston, Lincolnshire


### To Dr. BRADLEY.

Sir,

In the Medical Journal for May, I find a case of unusual impregnation and delivery, related by Mr. Cribb; if you think the following observations are anyways explanatory of so uncommon a circumstance, you will have the goodness to insert them in your next number.

I am, &c.

WILLIAM CRANE, M. D.

*Boston, Lincolnshire, Sept.* 22, 1805.

---

IT is much to be lamented, that Mess. Cribb and Cooper were not permitted to open the body after the death of their patient; from the well known accuracy and anatomical knowledge that these gentlemen possess, every particular of this extraordinary case would have been before your readers; though much as we may lament the want of such an inspection, we are greatly obliged to them for making the case public: the skilful manner in which they treated their patient, and the faithful account given by them of every circumstance, fully shew that they merit the high estimation that the neighbourhood in which they reside, have for many years entertained of their professional abilities.

In reasoning upon this case, to find an explanation for the menstrual flux and impregnation, we must have a strict attention to the facts before us; without indulging any any theory, and endeavour to account for them as near as possible, to the well known laws of Nature, we may certainly say, that the uterus, with its appendages, is absolutely necessary to either; that an opening outwards is equally so, as without which, neither the one nor the other could be accomplished. It is not necessary here to notice the various theories that have been formed to explain the cause of impregnation; as it seems perfectly agreed by all physiologists, that the rudiments of the new animal is seated in the ovarium of the female, and that by the proper application of a fluid destined for that purpose, it is in some peculiar manner excited into action, and evolves or produces a new creature of its own peculiar species.

The female, which is the subject of our present enquiries, had a child some years ago, and then had a bad labour, and was, as she expressed herself, very sore; before that confinement it does not seem that she was in any respect different from any other woman. From this fact, I presume, the whole will admit of an explanation; in her former labour I have no doubt but that a compleat laceration of the vagina and perinæum took place, which for want of proper care became ulcerated, and the acrid matter which it produced, corroded the rectum, and thus a communication was formed between that intestine and the vagina; from the same inattention prevailing, the injured perinæum and the vagina, with its labia, healed in a common adhesion, which all soft parts made sore will do, by the means of adhesive inflammation, or granulation. These parts healing in the manner here supposed, we can easily imagine that a fistulous communication was kept open between the rectum and vagina, through which the menstrual discharge took place. In regard to the impregnation which succeeded, I shall only say, that, to medical readers, I have said enough to be understood; and to those who are not of the medical profession, I do not wish to be more explicit; and if I do not mistake Mr. Cooper's meaning, in his letter to Mr. Cribb, he seems to have an opinion similar to mine.

Mr. Dawson, taking it for granted that Messrs. Cooper and Cribb, as well as Mr. Tice, were mistaken in the case, has argued with great ingenuity; but if that gentleman had the *honour* (as I have), of being personally acquainted with the two former of those gentlemen, he would have known, that they are the last, who are likely to be inaccurate, or to give a statement of any event, the truths of which they had not fully investigated. Mr. D. seems very tenacious of his opinion, and says, that he will not be convinced unless it be proved to him, how a woman could menstruate and conceive with an impervious vagina. This I have attempted; and if that gentleman forms an opinion different to mine, I shall not consider him as one of those that will not believe, though one should rise from the dead, therefore shall refer him to La Motte's Midwifry, Obs. 338, 339, 340; and to Philosophical Transactions, No. 237, page 56, where he will find, that whathas been described by Mr. Cribb, is an event that has been observed before, or at least very similar, and that it is possible for menstruation and impregnation to take place, although the vagina is not perforated.

